# Prevalence and associated risk factors of peste des petits ruminants in selected districts of the northern border region of Pakistan

**DOI:** 10.1186/s12917-024-04033-8

**Published:** 2024-05-24

**Authors:** Munibullah, Yanmin Li, Kainat Munib, Zhixiong Zhang, Zhidong Zhang

**Affiliations:** 1 College of Animal Husbandry & Veterinary Medicine, Southwest Minzu University, Chengdu, 610041 China; 2 Department of Clinical Studies, Faculty of Veterinary and Animal Sciences, Pir Mehr Ali Shah Arid Agriculture University, Rawalpindi 46000, Pakistan; 3 Department of Sociology, Allama Iqbal Open University, Islamabad, Pakistan; 4 Lanzhou Veterinary Research Institute, Lanzhou, 730046 China

**Keywords:** PPR, PPR-Virus, Epidemiology, Risk factors, Northern Pakistan

## Abstract

**Background:**

Peste des Petits Ruminants (PPR) is a world organization for animal health (WOAH) notifiable and economically important transboundary, highly communicable viral disease of small ruminants. PPR virus (PPRV) belongs to the genus *Morbillivirus* of the family *Paramyxoviridae*.

**Aim:**

The present cross-sectional epidemiological investigation was accomplished to estimate the apparent prevalence and identify the risk factors linked with peste des petits ruminants (PPR) in the previously neglected northern border regions of Pakistan.

**Method:**

A total of 1300 samples (serum = 328; swabs = 972) from 150 flocks/herds were compiled from sheep (*n* = 324), goats (*n* = 328), cattle (*n* = 324), and buffaloes (*n* = 324) during 2020–2021 and tested using ELISA for detection of viral antibody in sera or antigen in swabs.

**Results:**

An overall apparent prevalence of 38.7% (504 samples) and an estimated true prevalence (calculated by the Rogan and Gladen estimator) of 41.0% (95% CI, 38.0–44 were recorded in the target regions. The highest apparent prevalence of 53.4% (85 samples) and the true prevalence of 57.0%, 95% Confidence Interval (CI) were documented in the Gilgit district and the lowest apparent prevalence of 53 (25.1%) and the true prevalence of 26.0%, 95% Confidence Interval (CI), 19.0–33.0) was reported in the Swat district. A questionnaire was designed to collect data about associated risk factors that were put into a univariable logistic regression to decrease the non-essential assumed risk dynamics with a P-value of 0.25. ArcGIS, 10.8.1 was used to design hotspot maps and MedCalc’s online statistical software was used to calculate Odds Ratio (OR). Some of the risk factors significantly different (*P* < 0.05) in the multivariable logistic regression were flock/herd size, farming methods, nomadic animal movement, and outbreaks of PPR. The odds of large-sized flocks/herds were 1.7 (OR = 1.79; 95% Confidence Interval (CI) = 0.034–91.80%) times more likely to be positive than small-sized. The odds of transhumance and nomadic systems were 1.1 (OR = 1.15; 95% Confidence Interval (CI) = 0.022–58.64%) and 1.0 (OR = 1.02; 95% Confidence Interval (CI) = 0.020–51.97%) times more associated to be positive than sedentary and mixed farming systems, respectively. The odds of nomadic animal movement in the area was 0.7 (OR = 0.57; 95% Confidence Interval (CI) = 0.014–38.06%) times more associated to be positive than in areas where no nomadic movement was observed. In addition, the odds of an outbreak of PPR in the area were 1.0 (OR = 1.00; 95% Confidence Interval (CI) = 0.018–46.73%) times more associated to be positive than in areas where no outbreak of PPR was observed.

**Conclusions:**

It was concluded that many northern regions considered endemic for PPR, large and small ruminants are kept and reared together making numerous chances for virus transmission dynamic, so a big threats of disease spread exist in the region. The results of the present study would contribute to the global goal of controlling and eradicating PPR by 2030.

## Introduction

Peste des Petits Ruminants (PPR) is a world organization for animal health (WOAH) notifiable and economically important transboundary, highly communicable viral disease of small ruminants, which is characterized by severe morbidity and mortality rates [1]. PPR virus (PPRV) belongs to the only member of the Morbillivirus caprinae species within the genus *Morbillivirus* of the family *Paramyxoviridae*. There is only one serotype of PPRV, but phylogenetic analysis based on partial N or F gene sequences groups PPRV strains into lineages I, II, III, and IV. Lineage IV is currently most prevalent in Asian countries [2]. Clinically, PPR resembles rinderpest (RP) in cattle and is characterized by high fever, ocular-nasal discharges, necrotic stomatitis, and catarrhal inflammation of the ocular-nasal mucosa, enteritis, bronchopneumonia, and diarrhea followed by death or some time recovery from the disease [3]. The highest mortality and morbidity of disease are observed in small ruminants. The mortality ranges from 50 to 90% and sometimes can be nil and morbidity can be 10–100%, even lower than 10% depending on circumstances like general animal health status, immunity, previous exposure, nutritional condition and absence of secondary bacterial infection [4]. PPRV primarily affects sheep and goats; while cattle and buffaloes are infected asymptomatically with seroconversion, however camels and certain wild ruminants may show clinical signs, symptoms, and mortality [5]. PPR is endemic across Asia, the Middle East, and African regions. The widespread transmission of PPR across the world damages the livelihoods, food safety, and trade of herders as well as poses threats to biodiversity and ecological health [2]. As a result, PPR has pulled the consideration of FAO and WOAH and is listed as a major transboundary animal infection that needs to be prevented, controlled, and eradicated [6]. However, controlling PPR needs a good understanding of the epidemiological dynamics and the influence of the disease in a range of geographic regions and management structures [7, 8].

Throughout Asia where small ruminants contribute to assuring livelihoods, PPR is a main economic risk to the growth of sustainable animal production. The PPR in Asia was first described in southern India and currently remains endemic in many countries of Asia. In the Pan Pamir Plateau countries, PPR has caused significant economic damage to the animal production system and threatened wildlife. Various investigations showed that unrestricted transboundary animal movement as well as animal movement within a country is considered a major risk factor regarding the transmission of PPRV [9]. Recent study based on a MaxEnt model showed that five Least Cost Path (LCP) is responsible for PPRV cross-border transmission among China, India, Pakistan, Kazakhstan, and Tajikistan [10]. An epidemiological study showed that PPRV isolates caused the 2007 and 2013 PPRV epidemics in China was closely interrelated to lineage IV endemic bordering countries [11]. Epidemiological investigation and phylogenetic analysis of two distinct epidemics of PPR exposed that Pakistani isolate, collected with Chinese isolates, which are symbolic of the factual geographic pattern of PPRV [12]. Although PPRV introduction to China remains to be fully discovered, the PPR epidemics in China may be commenced by the cross-border spread of PPR from the neighboring enzootic states. Therefore, there should be usual migration tracks for domestic and wild animals and different associated risk factors near the western zones of China (N 29˚54’-44˚32’), which might assist the transboundary spread of PPRV due to contamination of grassland from various species sharing similar grazing points and having a status of no or irregular vaccination. These create a big issue in PPR eradication from the root level [10].

Pakistan occupies a location of great geostrategic significance, bordered by China on the northeast, Afghanistan on the northwest, Iran on the west, India on the east, and the Arabian Sea on the south. PPR is enzootic throughout Pakistan, where both small ruminants and large are mostly reared together within close interaction, regularly sharing bounded inhabitances as well as grassland and watering drinking points. These husbandry structures provide best chances for the spread of viruses among various sheep and goats populations as well as between small and large ruminants [13]. The current vaccination strategies of PPR in Pakistan and neighboring underdeveloped countries are insufficient and no proper vaccination policies, vaccine production facilities, or supply chain of vaccine to animal production systems. Furthermore, the huge gaps between local farmers, migratory nomadism/ transhumant, and concerned veterinary authorities, as well as regional policymakers, are existing. When considering eradication programs for PPRV in the future, these factors are of great significance, and without minimizing these gaps, there would be impossible to achieve the long-term goals of PPR eradication. Pakistan’s northern border is adjacent to Afghanistan, China, and Tajikistan border regions, but data on epidemiological dynamics and associated risk factors of PPR both in small and large ruminants of local and migratory flocks/herds are very scarce. The current study was conducted to estimate the apparent prevalence, identify the associated risk factors, and hotspot trends of PPR in the northern border regions of Pakistan which are previously neglected; conflict hit territories and having a significant geostrategic importance. The study will provide regional epidemiology, associated risk factors and GIS-based investigation of PPR and will identify that when and where intensive surveillance and immunization along with biosecurity procedures essential to be employed for the control and eradication of the infection from the research zones and adjacent neighbor countries in consonance with the PPR global control and eradication strategy.

## Materials and methods

### Study area

**Fig. 1:**
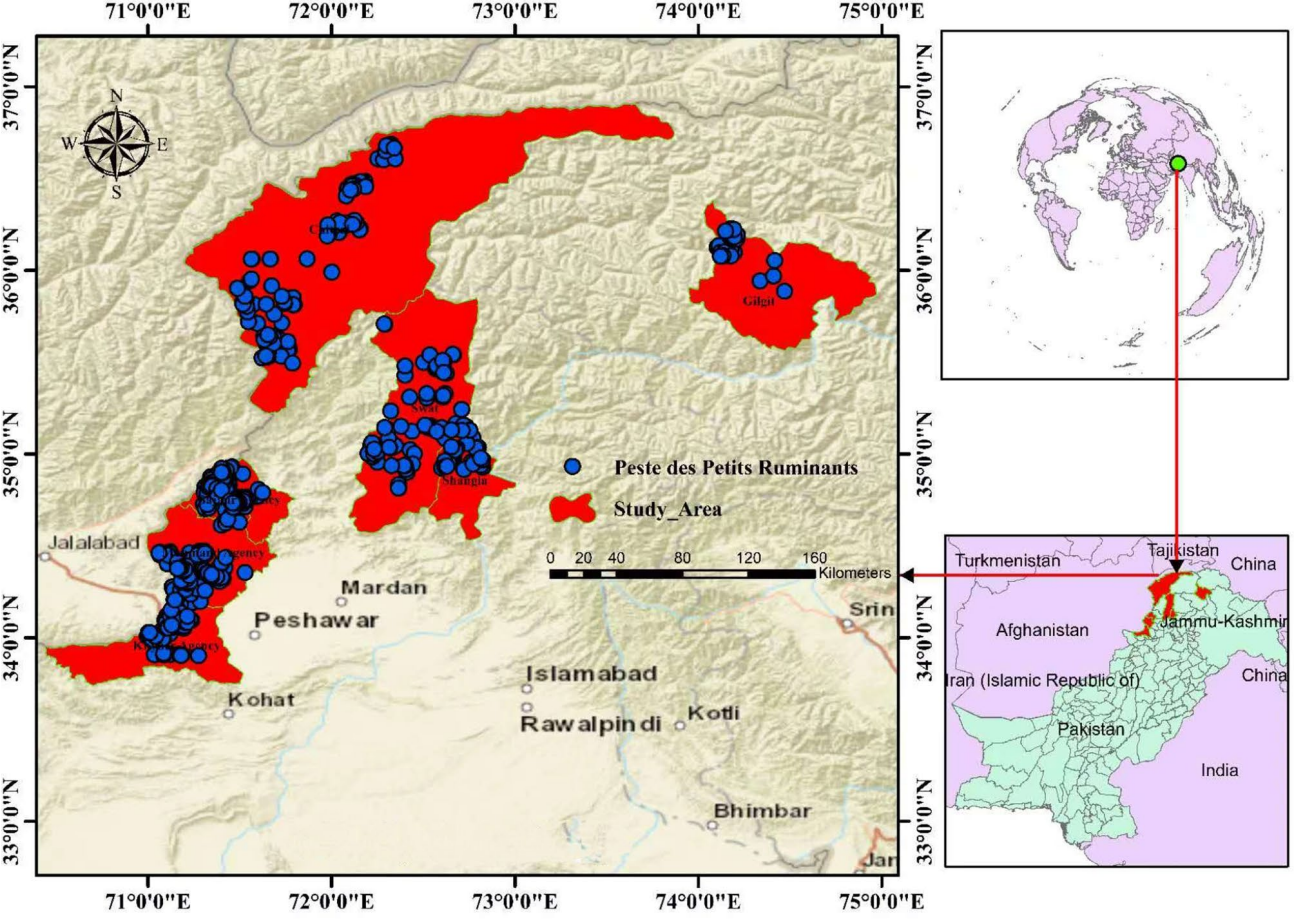
Map of Pakistan’s northern border regions showing the study sites and neighboring countries. The map was created using ArcGIS, 10.8.1 while drag GPS coordinates to the page and dropping it on the map drop zone by the author (Munibullah)

The study was conducted in Pakistan’s northern border, adjacent to Afghanistan, China, and Tajikistan border regions including Swat (35°12′N 72°29′E), Shangla (34°52′N 72°39′E), Chitral (35°50′N 71°47′E), Bajaur agency (34°41′N 71°30′E), Khyber agency (32°40′N 69°51′E), Mohmand agency (34°30′N 71°20′E), and Gilgit region (35°55′N 74°18′E) as shown in Fig. 1. The first three districts belong to the provincially administrated tribal areas (PATA) of Pakistan. The provincially administrated tribal areas (PATA) were the former administrative subdivision of Pakistan designated in Article 246(b) of the constitution of Pakistan. The remaining three agencies (Bajaur, Khyber & Mohmand) belong to the formerly federally administrated tribal areas (FATA). The FATA was a semi-autonomous tribal region in northwestern Pakistan, which existed from 1947 until being merged with the neighboring province of Khyber Pakhtunkhwa in 2018. It bordered Pakistan’s provinces of Khyber Pakhtunkhwa, Balochistan, and Punjab to the east, south, and southeast respectively, and Afghanistan’s provinces of Kunar, Nangarhar, and Paktia to the west and north. Furthermore, Gilgit is the capital town of Gilgit–Baltistan (GB) previously recognized as the Northern Areas of Pakistan. Gilgit is surrounded by the Wakhan corridor of Afghanistan in the north, the People’s Republic of China in the north and northeast, Skardu district in the south and southeast; Chitral is the northernmost district sharing a border with GB to the east, with Nuristan Badakshan, and Kunar provinces of Afghanistan to the west and north, and with the Dir and Swat districts of Khyber Pakhtunkhwa to the south, and a narrow band of Wakhan Corridor splits Tajikistan from Chitral in the north. Bajaur Agency (34°41′N 71°30′E) is located at a high altitude to the east of the Kunar region of Afghanistan and Pakistan, from which it is divided by a constant track of harsh boundary mountains, making a barrier that is easily travelable at one or two points; while Mohmand Agency is adjoined by Bajaur Agency to the north, Khyber Agency to the south, Malakand Agency and Charsadda district to the east and Peshawar to the southeast; Shangla is the district of Khyber Pakhtunkhwa, situated between the hillocks and surrounded by high mountains full of forests and green pastures; Khyber Agency is bordered by Nangarhar region of Afghanistan to the west, Kurram Agency to south west, Orakzai Agency to the south, Peshawar district to the east and Mohmand Agency in the north; Swat (35°12′N 72°29′E) is a natural geographic region of formerly provincially administrated tribal area of Khyber Pakhtunkhwa surrounding the Swat river having a green hills, forests and grazing pastures.

### Study animals

The target animals in Pakistan’s northwest and northeast border regions were cattle, buffaloes, sheep, and goats the study units were unvaccinated animals that are more than six months. Sheep and goats are classified under indigenous and cross breed, while, cattle were of Achai, Sahiwal, and crossbreed, on the other hand, buffalo were of Azakheli, Kundi, Nili-Ravi, and crossbreed.

### Determination of prevalence of PPR

#### Study design and sampling strategy

A cross-sectional study with 1300 samples were design to estimate the apparent prevalence and identify the associated risk factors of peste des petits ruminants (PPR) in the previously neglected northern border regions of Pakistan during 2020–2021.

#### Collection of blood and swab samples

Blood samples (about 5 ml) was collected right from the jugular vein of animals by venipuncture using a sterile syringe and needles and then transferred to a labeled gel-barrier tube with an identification code. The blood samples were kept at a slant position overnight at room temperature. A clear serum was poured into an Eppendorf tube (2 ml) and categorized consequently and stored in the freezer till its arrival at the laboratory and stored in a freezer (-20 °C). Similarly, swab samples from nasal, ocular, oral, and rectal regions were collected and kept in the refrigerator until arrival at the laboratory and stored. All serum and swab samples were shipped to the Veterinary Research Institute, Peshawar, Pakistan by keeping the cold chain and, and stored at − 20 °C. All the samples were collected from randomly selected different animals for swab and for serum samples.

#### Competitive ELISA for detection of the antibody to PPRV

The serum samples were tested using PPR competitive ELISA (cELISA) kit according to the instructions of Anderson et al. [14], and the manufacturer (Lanzhou Veterinary Research Institute, CAAS Xujiaping No. 1 Laznzou, Gansu, 730,046, (patent no: ZL201210278970.9) for detection of PPPV antibody. The inhibition rate (PI value) of each sample was calculated according to the following formula: PI= [1-(sample’s OD_450_/MAb OD_450_)] ×100%. The experiment was only tenable when the negative control serum PI < 40%, positive control serum PI > 60%, and blank control PI ≥ 90%. For evaluating the antibody titer in sera, when the PI ≥ 45%, the serum was positive; when PI < 40%, the serum was negative; when 40% < PI < 45%, the serum was then suspicious. In the diagnosis or epidemiological investigation, when PI is greater or equal to ≥ 50%, the serum was positive; when PI is less than 50%, the serum was a negative.

#### Sandwich ELISA for detection of PPRV antigen

Swabs samples collected were tested using PPR antigen capture sandwich ELISA Kit “ID. Vet France” was used to detect according to the manufacturer’s instruction. To interpret the results for each sample the S/P% is calculated. S/P %= (OD sample-ODNC)/ (ODPC - ODNC) ×100. Where PC: positive control; NC: negative control. If the S/P% is less than 20%, then the sample was considered negative. If the S/P% is greater or equal to 20%, the sample was considered positive.

### Data collection

Data collection adapted participatory epidemiological (PE) appraisal techniques for gathering of disease epidemiological data as described by Catley et al. [15]. Participatory epidemiological approaches based on open communication and transfer of knowledge, using a toolkit of methods guided by some key concepts and attitudes regarding disease under investigation and regional geostrategic scenario. The methods include: A semi-structured interviewing and collection of information through structured questionnaire about animal species, sex, age, flock size, grazing dynamics, farming method, vaccination status, the entry of new animals into a herd/ flock, information about the outbreak of PPR, nomadism & cross-boundary movements, returned of unsold animals into the flock/herd, the appearance of the clinical sign of PPR and availability of nearby veterinary services, focus-group discussions, ranking and scoring disease observations, a variety of visualization (e.g. mapping and modelling) and diagramming techniques (e.g. seasonal calendars and historical timelines regarding PPR in the region), all information was validated by cross-checking. The geographical positioning system (GPS) coordinates were obtained in the form of latitudes and longitudes. The size of the flock/herd was categorized as less than 50, 51–100, 101–200, 201–300, 301–500, and more than 500. The age of animals was noted through physical observation, dentition, and inquiring from the farmers. The age was classified as less than 1 year, 1 year, 2 years, 3 years, and above 3 years.

#### Data management and analysis

Data were entered into the MS excel spreadsheet 2020 program, coded, and transferred to the Statistical Package for Social Sciences (SPSS) version 20. Overall apparent prevalence was estimated by Thrusfield [16]. The formula is given by:

Apparent prevalence= (positive samples /total number of animals sampled) x100.

The apparent prevalence estimates were used to estimate the true prevalence using Rogan and Gladen estimator [17]. The formula is given by:

$$Ture{\text{ }}prevalence = Ap + Sp - 1/Se + Sp - 1.$$

Where AP is apparent prevalence and Sp and Se are test specificity 99.2% and sensitivity 100% respectively for sandwich ELISA ID. vet (France). While a Competitive Enzyme Linked Immuno-Sorbent Assay (cELISA: Chinese Patent No. ZL201210278970.9) supplied by the Lanzhou Veterinary Research Institute kit has a has a diagnostic specificity (Sp) and diagnostic sensitivity (Se) of 97.7% and 84%, respectively, according to LVRI‐CAAS (176 sera tested) in comparison to the commercial ID Screen® PPR Competition ELISA (ID-Vet, France) [17, 18]. The Epi-Info online software (version 3.5.1) was used to calculate the confidence interval for proportions. Univariable logistic regression analysis for the proportions was carried out with *P* = 0.25. Multi-collinearity of risk factors was checked. This was verified additional by multivariable logistic regression analysis for the decision with a probability predictive limit of less than 5%. The MedCalc’s online statistical software was used to calculate Odds Ratio (OR) to associate the statistical power of PPR positivity with various possible risk factors. The interaction consequence of significant risk dynamics in the multivariable logistic regression analysis was also assessed. Model fitness was calculated by applying the Hosmer-Lemeshow goodness of the test (P value > 0.05).

ArcGIS 10.8.1 was used to design hotspot maps from GPS coordinates to highlight the location of each case in their localities.

## Results

### Prevalence of PPR

A total of 1300 samples were collected from 150 conveniently selected flocks/herds, including 328 serum samples and 972 swab samples from different animals. The serum samples and swab samples were tested by cELISA for antibody detection and sandwich ELISA for viral antigen detection, respectively. Out of a total of 328 serum samples analyzed by cELISA, 167 (50.09%) were positive for PPRV antibody. Out of a total of 972 swabs tested by sandwich ELISA, 337 (34.6%) were positive for PPRV antigen. Based on the detection rate of PPRV antibody and antigen, an overall apparent prevalence and true prevalence of 38.7% and 41.0% respectively, at 95% Confidence Interval (CI) was recorded in the target region (Table 1).

**Table 1:** The apparent prevalence and true prevalence of PPRV antibody or antigen in different types of samples using cELISA and sandwich ELISA.

Sample types	Number of samples	PPRV antibody (cELISA)		PPRV antigen (sandwich ELISA)		AP TP (95% Confidence Interval (CI)	
		*P*	*N*	*P*	*N*		
Serum	328	167	161	*	*	50.9%	58.0% (51.0–64.0)
Nasal	352	*	*	145	207	41.1%	41.0% (36.0–46.0)
Ocular	281	*	*	83	198	29.5%	29.0% (24.0–35.0)
Oral	224	*	*	75	149	33.4%	33.0% (27.0–40.0)
Rectal	115	*	*	34	81	29.5%	29.0% (21.0–39.0)
Total	1300	total P = 504; total N = 796				38.7%	41.0% (38.0–44.0)

* Not applicable, AP = apparent prevalence, TP = true prevalence

#### The district-wise prevalence of PPR

The district-wise prevalence of PPR was analyzed based on the positive rate of PPRV antibody and antigen. As shown in Table 2, out of the seven regions studied, the highest apparent prevalence of 53.4% and true prevalence of 57.0% (95% CI = 48.0–66.0%) was documented in the Gilgit region, followed by Chitral, Bajaur Agency, Mohmand Agency, Shangla, Khyber Agency and Swat district.

**Table 2:** The district-wise prevalence of PPR based on a combination of antibody and antigen ELISA

Locations	Number of tested samples	Positives	Apparent prevalence (%)	True prevalence 95% Confidence Interval (CI)
Mohmand Agency	220	85	38.6%	41.0 (34.0–48.0)
Khyber Agency	200	61	30.5%	32.0 (25.0–39.0)
Bajaur Agency	184	79	42.9%	45.0 (37.0–54.0)
Swat	211	53	25.1%	26.0 (19.0–33.0)
Chitral	161	81	50.3%	54.0 (45.0–63.0)
Gilgit	159	85	53.4%	57.0 (48.0–66.0)
Shangla	165	60	36.3%	38.0 (30.0–47.0)

#### The species-wise prevalence of PPR in the target region

The species-wise apparent prevalence of PPR was analysed based on data from cELISA and sandwich ELISA. A total of 324 sheep, 328 goats, 324 buffaloes, and 324 cattle were sampled and an apparent prevalence of 52.1%, 51.8%, 27.4% and 23.4% was recorded, respectively. However, a true prevalence of 56.0% (95% Confidence Interval (CI) = 50.0–62.0), 55.0% (95% Confidence Interval (CI) = 49.0–62.0), 28.0% (95% CI = 23.0–34.0) and 24.0% (95% Confidence Interval (CI) = 19.0–29.0) was estimated for sheep, goats, buffaloes, and cattle respectively. The highest prevalence was observed in the sheep and goat population and the lowest was observed in the cattle population shown in (Table 3).

**Table 3:** The species-wise prevalence of PPR based on a combination of antibody and antigen ELISA

Species	Number of tested samples	Positives	Apparent prevalence %	True prevalence 95% Confidence Interval (CI)
Sheep	324	169	52.1%	56.0 (50.0–62.0)
Goat	328	170	51.8%	55.0 (49.0–62.0)
Cattle	324	76	23.4%	24.0 (19.0–29.0)
Buffalo	324	89	27.4%	28.0 (23.0–34.0)

### Analysis and assessment of risk factors

#### Univariable logistic regression analysis of risk factors for PPR positivity in animals

Univariable logistic regression was used to analyze risk factors associated with PPR positivity in sheep, goats, cattle, and buffaloes. Various factors omitted from the model by applying univariable logistic regression analyses with a p-value of 0.25 were age, sex, introducing new animals, type of flock/herd, and season. Accordingly, the univariable logistic regression analysis and multivariable logistic regression analysis specie (*P* = 0.000), flock/herd size (*P* = 0.004), outbreaks of PPR or PPR-affected animals in the area in the last 15 days (*P* = 0.000), nomadic animals’ movement (*P* = 0.000), farming methods (*P* = 0.021), return of unsold animals from the market (*P* = 0.057) and outbreak location (*P* = 0.046) were significantly risk factors for the occurrence and distribution of PPR in the target region (Table 4).

#### Risk assessment

**Fig. 2:**
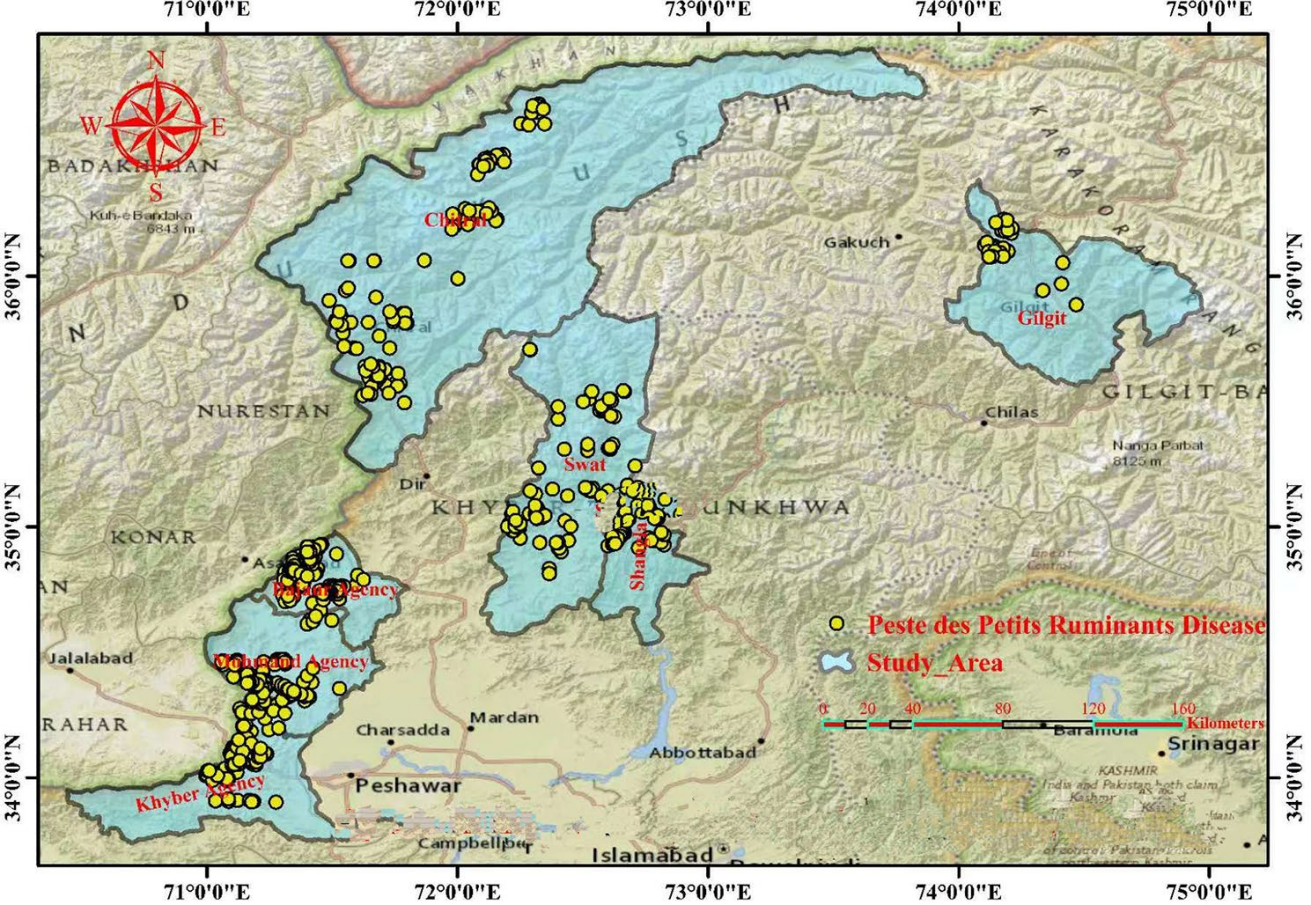
Hotspot map of the spatial distribution of PPR. The map identified seven PPR disease hotspots trend categories across different sub-regions in Pakistan’s northern border region based on participatory epidemiological tools and PPR positivity. The greenish zones represent study areas while the yellow spots show the burden of PPR cases in different study districts (Mohmand, Khyber, Bajaur, Swat, Chitral, Gilgit, and Shangla). The map was created using ArcGIS, 10.8.1 while drag GPS coordinates to the page and dropping it on the map drop zone by the author (Munibullah)

The assessment of risk factors associated with PPR positivity in the region was evaluated as a function of the probability of hazard (PPR) calculated positivity (Table 2), exposure of susceptible usual hosts (sheep, goats) and unusual hosts (cattle and buffaloes) shown in Tables 3 and 4 and the consequences of spread of PPR using the following parameters: current true prevalence and relevant odds ratios of infection (Tables 2 and 4), evidence of unvaccinated nomadism and transboundary animal movements shown in Table 4, cELISA and sandwich ELISA screening records of an infected animals through different types of samples (Table 1), the outbreak of PPR or PPR-affected animals in the area in the last 15 days and the virus potential for infection in the entire region as shown in Fig. 2; Tables 2 and 4 with *P* < 0.05, and other findings provide a strong sero-epidemiological foot printings of PPR endemic dynamics and transboundary threats in the region. Based on current risk assessment across many northern regions considered endemic for PPR, large and small ruminants are kept and reared together making numerous chances for virus transmission dynamic. This is the first assessment of PPRV positivity in small and large ruminant populations in the northern border region of Pakistan adjacent to Afghanistan, Tajikistan, and China border regions based on the epidemiological foot printing of the animals sampled.

**Table 4:** Univariable logistic regression analyses of risk factors for PPR positivity

Variables	Cluster	Tested samples	Positives	Apparent Prevalence in % 95% Confidence Interval (CI)	Odds Ratio 95% Confidence Interval (CI)	*P*-value
Species	Sheep	324	169	52.1 (46.7–57.6)	1.06 (0.021–53.88)	0.000
	Goat	328	170	51.8 (46.4–57.2)	1.07 (0.021–54.45)	
	Cattle	324	76	23.4 (18.8–28.0)	0.30 (0.006–15.64)	
	Buffalo (ref)	324	89	27.4 (22.6–32.3)	0.38 (0.075–19.29)	
Age	< 1 year	70	30	42.8 (31.2–54.4)	0.75 (0.014–39.03)	0.641
	1 year	130	48	36.9 (28.6–45.2)	0.58 (0.011–30.10)	
	2 year	531	201	37.8 (33.7–41.9)	0.60 (0.012 -30.84)	
	3 year > 3 year (ref)	444 123	181 44	40.7 (36.2–45.4) 35.7 (27.3–44.2)	0.68 (0.013–34.87) 0.55 (0.010 -28.69)	
Sex	Male	277	119	42.9 (37.1–48.7)	0.75 (0.014–38.27)	0.686
	Female (ref)	1023	385	37.6 (34.6–40.6)	0.60 (0.012–30.49)	
Flock/Herd size	< than50 51–100	353 663	159 199	45.0 (39.8–50.2) 30.0 (26.5–33.6)	0.82 (0.016–41.56) 0.42 (0.008–21.72)	
	101–200	126	81	64.2 (55.9–72.6)	1.79 (0.034–91.80)	0.004
	201–300	115	55	47.8 (27.9–43.0)	0.91 (0.017–47.02)	
	301–500	28	5	17.8 (3.6–32.0)	0.23 (0.004- 13.14)	
	> than 500 (ref)	15	5	33.3 (9.4–57.1)	0.52 (0.009–30.17)	
Vaccination status	Non-vaccinated	1265	482	38.1 (35.4–40.8)	0.61 (0.012–31.08)	1.000
	Irregular vaccinated	35	12	34.2 (10.8–59.8)	0.53 (0.009–28.45)	
The outbreak of PPR or PPR-affected animals in the area in the last 15 days	Yes	556	267	48.0 (43.8–52.1)	1.00 (0.018–46.73)	0.000
	No	744	237	31.8 (28.5–35.2)	0.46 (0.009–23.65)	
Introduced new animals	Yes	446	200	44.8 (40.2–49.4)	0.81 (0.016–41.17)	0.607
	No (ref)	854	304	35.5 (32.3–38.8)	0.55 (0.010–27.94)	
Nomadic animals movement in the area in the last 15 days	Yes	917	394	42.9 (39.7–46.1)	0.75 (0.014–38.06)	0.000
	No (ref)	383	110	28.7 (24.1–33.2)	0.40 (0.008–20.49)	
Farming methods	Sedentary	383	139	36.2 (31.4–41.1)	0. 57(0.011–28.91)	
	Transhumance	250	134	53.6 (47.4–59.7)	1.15(0.022–58.64)	0.021
	Nomadic	257	130	50.5 (44.4–56.6)	1.02 (0.020–51.97)	
	Mixed (ref)	410	101	24.6 (20.4–28.8)	0.32 (0.006–16.63)	
Clinical sign of PPR	Yes	173	149	86.1 (80.9–91.2)	6.10 (0.1183–314.7)	0.000
	No	1127	355	31.4 (28.7–34.2)	0.46 (0.009–23.24)	
Type of flock/herd	Sheep	265	135	50.9 (44.9–56.9)	1.03 (0.020–52.71)	0.467
	Goat	262	136	51.9 (45.8–57.9)	1.07 (0.021–54.79)	
	Cattle	294	51	17.3 (13.0–21.6)	0.21 (0.004–10.78)	
	Buffalo	310	86	27.7 (22.7–32.7)	0.38 (0.007–19.57)	
	Mixed (ref)	169	96	56.8 (49.3–64.2)	1.13 (0.025–66.95)	
Return of unsold animals from the market	Yes	168	48	28.5 (22.3–36.2)	0.40 (0.007–20.57)	0.057
	No	1132	456	40.2 (37.4–43.1)	0.67 (0.013–34.07)	
Outbreak location	Mohmand Agency	220	85	38.6 (32.2–45.0)	0.63 (0.012–32.10)	0.046
	Khyber Agency	200	61	30.5 (24.1–36.8)	0.44 (0.008–22.47)	
	Bajaur Agency	184	79	42.9 (35.7–50.0)	0.75 (0.014–38.39)	
	Swat	211	53	25.1 (19.2–30.9)	0.33 (0.006–17.22)	
	Chitral	161	81	50.3 (42.5–58.0)	1.01 (0.019–51.64)	
	Gilgit	159	85	53.4 (45.7–61.2)	1.14 (0.022–58.55)	
	Shangla (ref)	165	60	36.3 (29.0–43.7)	0.57 (0.011–29.27)	
Seasons	Jan-March	404	152	37.6 (32.9–42.3)	0.60 (0.011–30.59)	0.551
	April-June	504	195	38.6 (15.4–22.2)	0.63 (0.012–31.96)	
	July-Sep	174	75	43.1 (35.7–50.4)	0.75 (0.014–38.29)	
	Oct-Dec (ref)	218	82	37.6 (31.1–44.0)	0.60 (0.011- 30.75)	

#### Association of clinical signs/symptoms with PPR in sheep and goats

The relationship/association between clinical signs and symptoms with PPR specifically in sheep, goats excluding cattle and buffaloes, was determined using co-efficient values, which were interpreted as follows: 0.0-0.199 for a very weak/no association, 0.2–0.39 for a weak association, 0.4-0.599 for a moderate association, 0.6-0.799 for a strong association, and 0.8-0.999 for a very strong association [19]. It was observed during field epidemiological investigation and interaction with farming communities that the disease primarily affects sheep and goats; however large ruminants were infected asymptomatically with seroconversion. It is indicated from the analyzed data (Table 5) that some of the signs and symptoms of the disease are weakly associated with PPR. On the other hand, some of the clinical signs and symptoms have a moderate association/relationship with PPR.

**Table 5:** Association of clinical signs/symptoms with PPR

Sign symptoms	Status	Positive (*n* = 504)	Percent%	Contingency co-efficient value	Association	
Temperature	Yes	265	52.5%	0.485	Moderate	
	No	239	47.5%			
Ocular discharge	Yes	199	39.4%	0.425	Moderate	
	No	305	60.5%			
Mucopurulent nasal discharge	Yes	189	37.5%	0.423	Moderate	
	No	314	62.4%			
Increase respiration rate	Yes	281	55.7%	0.501	Moderate	
	No	223	44.2%			
Increase pulse rate	Yes	278	55.2%	0.495	Moderate	
	No	225	44.7%			
Increase (CRT) capillary refill time	Yes	277	54.9%	0.500	Moderate	
	No	227	45.0%			
Diarrhea	Yes	155	30.7%	0.287	Weak	
	No	349	69.2%			
Anorexia	Yes	289	57.3%	0.428	Moderate	
	No	215	42.6%			
Dullness	Yes	298	59.1%	0.376	Weak	
	No	206	40.8%			
Depression	Yes	296	58.7%	0.378	Weak	
	No	208	41.2%			
Abortion	Yes	98	19.4%	0.334	Weak	
	No	406	80.5%			

### Hotspots of the spatial distribution of PPR

On spatial epidemiological investigation of outbreak records, PPR risk hotspots showed a wide deviation in the various regions of northwestern and northeastern Pakistan at different periods. Most of the study regions were considered as neglected areas of disease investigation including PPR. Therefore, special attention was taken to monitors the nomadic flock/herds and participating study activities in harsh full conditions. Fig. 2 (the map created using ArcGIS, 10.8.1) shows the spatial distribution of PPR in selected neglected areas of Pakistan’s northern border regions based on disease coordinates (latitudes and longitudes). The seven PPR disease hotspots trend categories were identified across different sub-regions in Pakistan’s northern border region based on both the detection rate of PPRV antibody and antigen. The greenish zones represent study areas while the yellow spots show the burden of PPR cases in different study districts. These disease hotspots were identified through the tools of participatory epidemiological (PE) assessment as discussed by Jost et al. [20], and Catley et al. [15], among visualization techniques; seasonal calendars, mapping, and diagramming exercises were the most common. Participatory mapping was one of the most useful tools in the PE toolkit, and was often a good technique to start with, as it involves several people and can stimulate much informal interviews and focused group discussion and enthusiasm. It was used to gain an overview of the spatial distribution of community resources, herding patterns, livestock population contact structure, the spatial distribution of risk factors, similarly questionnaire-based surveys, evidence of traditional routes of transboundary animal movements to these areas, seasonal migration within the country and across the border to these regions, livestock practices without vaccination, and based on current results, which shows that the disease is prevalent in these regions and act as a continue spreading points locally and regionally. In participatory epidemiological approaches, participatory mapping was used to map disease outbreaks, both spatially and temporally, within target communities. Respondents indicate the locations and dates of clinical disease events and describe the sequence of events, which reflects how diseases spread through communities and populations. This can highlight key risk factors and important epidemiological information, as well as contribute data to aid in estimating transmission parameters for disease models. It is a proven technique that overcomes many of the limitations of conventional epidemiological methods and has been used to solve several animal health-related events investigation and research problems. The approach can be developed in small-scale, community animal health programs, and also can be applied to major regional and international disease control efforts [19].

## Discussions

Though a variety of studies have been taken regarding PPR globally, the current study contributes to FAO/WOAH’s goal of achieving global PPR eradication in the future, by controlling the disease in the previously neglected or conflict-hit territories where the evidence of small ruminants–large ruminants and livestock-wildlife interfaces exist. The presence of the PPRV among unvaccinated animals in the study area was demonstrated by the clinical picture [21], sandwich ELISA and the PPRV-specific antibodies were detected using cELISA during 2020–2021. There was no recognized standard information on prior immunization in the study area; therefore, the existence of PPR antibodies was attributed to natural PPR infection.

It is evident (Table 2) that PPR is prevalent throughout the study region. The overall apparent prevalence based on both the detection rate of PPRV antibody and antigen in different animal species in the current study was 38.7% (*n* = 1300) of which 52.1% (*n* = 324) was detected in sheep, 51.8% (*n* = 328) in goats, 27.4% (*n* = 324) in buffaloes and 23.4% (*n* = 324) in cattle, similarly, the estimated true prevalence of PPR in the target region was 41.0% (95% Confidence Interval (CI) = 38.0–44.0), of which 56.0% (95% Confidence Interval (CI) = 50.0–62.0) was detected in sheep, 55.0% (95% Confidence Interval (CI) = 49.0–62.0) in goats, 28.0% (95% Confidence Interval (CI) = 23.0–34.0) in buffaloes and 24.0% (95% Confidence Interval (CI) = 19.0–29.0) in cattle (Tables 3 and 4). Regarding the detection of the PPR virus in large ruminants [13] and 23 of 250 (9.2%; 95% Confidence Interval (CI) = 5.9–13.5%) yaks sampled in Pakistan were found positive [22], are in line with the findings of current investigation. Furthermore, the retrospective studies in Pakistan excluding the current study area, showed a prevalence of 43.33% and 59.09% in small ruminants and large ruminants respectively [23] and 74.9% in sheep and goats [24]. Similarly, a Food and Agriculture Organization (FAO) project (GCP/PAK/127/USA) Progressive control of Peste des Petits Ruminants in Pakistan supported study investigated antibodies in the serum using cELISA and antigen in the tissue samples using IcELISA from 62 outbreaks against the PPR virus with a positive percentage of 61.27% and 64.99% respectively [25]. These studies are in line with the finding of the current investigation. As compared to the current study outcome, a higher overall apparent prevalence was documented in various Asian countries 74.9% in Pakistan Zahur et al. [24], 67.9% in India Saritha et al. [26], 48% in Afghanistan Azizi et al. [27], and 9.2% in yaks Abubakar et al. [22] 10.0% in cattle and 14.16% in buffaloes in Pakistan Abubakar et al. [13] were lower prevalence recorded as compared to the outcomes of the present study. The dissimilarities in the PPRV prevalence found in bordering nations compared to the present study region might be due to variations in the livestock management practices, seasonal variations, host population, sampling procedures used, disease control strategies, or practical data levels of natural protection and variable usual PPRV infection rates in various geographic regions.

Geographical regions that recorded the highest prevalence of PPR were Gilgit (53.4%) and Chitral (50.3%) while Swat (25.1%) had the lowermost PPR prevalence. The higher PPR positivity in Gilgit and Chitral can be described by rigorous unrestrained transboundary animal movements between these territories with Kunar, Badakhshan, and Nuristan provinces of Afghanistan, the Wakhan corridor of (Tajikistan-Afghanistan) in the north, the People’s Republic of China in the northeast and north where PPR epidemics have been observed in the past. Furthermore, poor livestock management practices and the use of mutual grazing structures, and huge nomadism could also contribute to this higher positivity. The climatic factors, livestock, domesticated yokes, and wildlife interface in pastoralist systems particularly in the regions with a high mass of wild animals like Gilgit and Chitral might lead to the highest PPR positivity. These outcomes are consistent with the studies of Noman et al. [28], suggesting that high rainwater and cold climate might lead to PPR spread. Limited data existing on PPR transmission from wild to domestic and from small ruminates to large ruminants in the study regions. Furthermore, these outcomes are consistent with the findings of Gao et al. [10], who investigated unknown regions of PPR transmission, further, the internal threat in China is lower than that in Pan Pamir Plateau states, also investigate, five representing corridors (Table 1), and verifies the probability of transboundary spread of PPR for the first time by small ruminants, large ruminants, and wild animals. In the FATA region of the Khyber Pakhtunkhwa province, the highest positivity of PPR was observed in Bajaur (42.9%), Mohmand (38.6%), and Khyber (30.5%), while (36.2%) prevalence in shangla district was reported. The maximum PPR positivity in these regions might be described by exhaustive unrestrained nomadism, climate factors, negligence in vaccination, and war conflicts among different tribes and nations.

There are several production systems while farming animals in the region, namely, nomadic, transhumant, sedentary, and household/mixed. Mostly small ruminants are raised in the nomadic and transhumant production systems [29]. Animals reared in a joint production structure like nomadic, transhumance, and/or free-grazing husbandry were probable to have higher prevalence with corresponding prevalence levels of 53.6% and 50.5%, whereas the lower prevalence of 24.6% was found in the animals of the mixed farming system. The odds of transhumance and nomadic farming system were 0.59 (OR = 0.595; 95% Confidence Interval (CI) = 0.306–1.158%) and 0.51 (OR = 0.519; 95% Confidence Interval (CI) = 0.272–0.900%) times more associated to be positive than sedentary and mixed farming systems, respectively with (*P* = 0.001). The odds of nomadic animal movement in the area in the last 15 days was 0.5 (OR = 0.552; 95% Confidence Interval (CI) = 0.389–0.784%) times more associated to be positive than in areas where no nomadic movement was observed. The outcomes of the present study are inconsistent with the outcomes of Zahur et al. [25]. Furthermore, a huge transboundary animal movement from Afghanistan via the Khyber agency, Mohmand agency, Bajaur agency, and Tajikistan via the Wahkan corridor and adjacent border regions were investigated through participatory epidemiological discussion with the local communities, these observations are in line with the findings that these nomads visit different areas, especially riverbanks, irrigated areas of Khyber Pakhtunkhwa, Punjab and Sindh provinces of Pakistan in the winter season, and northern border regions of Pakistan in the summer season. In wheat harvesting season, these nomads came back to Afghanistan adopting the same paths [25, 26]. These are the main epidemiological footprints behind the endemic status of PPR virus circulation in the study region.

The logistic regression model indicated that the odds of large-sized flocks/herds (101–200) of animals were 1.7 times more positive than small-sized flocks/herds. This finding is in agreement with Selvaraju [30], . Table 4 indicated that large-sized flocks/herds of animals were 1.7 times significantly more at risk (*P* = 0.004), of getting PPR infection (OR = 1.79; 95% Confidence Interval (CI) = 0.034–91.80). The odds of medium-sized herds/flocks of animals being positive was 0.42 times more likely than small herds/flocks OR = 0.42; 95% Confidence Interval (CI) = 0.008–21.72) shown in (Table 4) shows that overcrowding might be a contributing factor in the spread of the contagious PPR virus among susceptible animals. This judgment is consistent with Al-Majali et al. [31]. This overcrowding might increase the spread of the contagious PPR virus among susceptible animals [32].

The outcomes of the present investigation indicated that the introduction of new animals into a flock/herd in the last 15 days showed a greater positivity of 44.8% (95% Confidence Interval (CI) = 40.2–49.4%), (Table 4). This result is in line with the studies of Gebre et al. [33]. After buying animals, owners do not follow isolation practices. Animals are taken to the market and brought home on foot crossing long distances. During this stressful time, the animals become susceptible to different infections. Further, when animals from various stocks are together in one marketplace and there may be interactions. Subsequently, this phenomenon plays an important role in the PPRV transmission dynamic [32].

Associated risk factors that were statistically non-significant with the positivity of PPR in the region, were age (*p* > 0.05), sex (*p* > 0.05), the introduction of new animals into the flock or herd in the last 15 days (*p* > 0.05), type of flock/herd (*p* > 0.05), return of unsold animals from the market (*p* > 0.05), and seasons (*p* > 0.05). However, slightly high PPR apparent prevalence (52.1%) and a true prevalence of 56.0% (95% Confidence Interval (CI) = 50.0–62.0) in sheep than in goats (51.8%) and 55.0% (95% Confidence Interval (CI) = 49.0–62.0) in the study region. Similarly, the higher prevalence of PPR in buffalo (27.4%) than in cattle (23.4%) with most of them infected asymptomatically the current findings agrees with the outcomes of Khan et al. [32] who documented a significantly higher prevalence of 67.42% in buffalo and 41.86% in cattle with (*P* = 0.005), Abubakar et al. [13] who documented a significantly higher prevalence of 14.16% in buffaloes and 10.0% in cattle in Pakistan and of Balamurugan et al. [1] who detected a slightly higher seroprevalence of 16.20% in buffaloes and 11.07% in cattle, across 1498 serum samples analyzed in the neighboring country, India. On the other hand, this finding is dissimilar from the outcomes of Saritha et al. [26], and Kgotlele et al. [34], who reported higher prevalence in goats than sheep. Similarly, in contrast to the current study lower prevalence of 5.88% PPRV antibodies in cattle was reported by Prajapati et al. [35] in Nepal.

The study identified seven PPR disease hotspots trend categories across different sub-regions in Pakistan’s northern border region. It was concluded that no immunization, the practice of introducing newly purchased animals, congestion, the presence of PPR-affected animals in the area, nomadism, and transboundary movements were the main associated risk factors of disease occurrence in the region, and the hotspots map showed that big threats of disease spread exist to neighbor’s states and vice versa. Across many northern regions considered endemic for PPR, large and small ruminants are kept and reared together making numerous chances for virus transmission dynamic. This study provides a spark for policymakers regarding regional and global goal achievements of PPR eradication by 2030.

## Data Availability

The authors declare they have no competing interests. The datasets generated and/or analyzed during the current study are not publicly available due to the confidentiality agreements made all authors, but could be available from the corresponding author on reasonable request.

## References

[1] Balamurugan V, Krishnamoorthy P, Raju DS, Rajak KK, Bhanuprakash V, Pandey AB, Gajendragad MR, Prabhudas K, Rahman H (2014). Prevalence of Peste-Des-petits-ruminant virus antibodies in cattle, buffaloes, sheep and goats in India. Virusdisease.

[2] Md A. (2020). Peste des petits ruminants (PPR) in Africa and Asia: A systematic review and meta-analysis of the prevalence in sheep and goats between 1969 and 2018. Veterinary Medicine and Science, 6 (4):813–33. 10.1002/vms3.300 PMID: 32529792. 10.1002/vms3.300 PMC7738735 32529792

[3] Gargadennec L, Lalanne A (1942). La Peste Des petits ruminants. Bull Serve Zootech Epizoot Afr Occid Fr.

[4] Abu-Elzein EME, Hassanien MM, Al-Afaleq AI, Abd-Elhadi MA, Housawi FMI (1990). Isolation of peste des petits ruminants from goats in Saudi Arabia. Vet Rec.

[5] Albina E, Kwiatek O, Minet C, Lancelot R, Servan de Almeida R, Libeau G (2013). Peste Des petits ruminants, the next eradicated animal disease?. Vet Microbiol.

[6] Mousumi B, Raja WY, Pronab D, Rabindra PS. (2018). An overview of process intensification and thermo stabilization for upscaling of Peste des petits ruminants vaccines in view of global control and eradication. Virusdisease, 29(3):285–96. 10.1007/s13337-018-0455-3 PMID: 30159362. 10.1007/s13337-018-0455-3 PMC6111956 30159362

[7] Munibullah, Li Y, Munib K, Zhang Z (2022). Regional epidemiology and associated risk factors of PPR in Asia-A Review. Slovenian Veterinary Res.

[8] Akwongo CJ, Quan M, Byaruhanga C (2022). Prevalence, risk factors for exposure, and SocioEconomic Impact of Peste Des Petits Ruminants in Karenga District, Karamoja Region, Uganda. Pathogens.

[9] Munir M. (2013). Role of Wild Small Ruminants in the Epidemiology of Peste Des Petits Ruminants. Transboundary and Emerging Diseases, 61(5):411–24. 10.1111/tbed.12052 PMID: 23305511. 10.1111/tbed.12052 23305511

[10] Gao S, Xu G, Zeng Z, Lv J, Huang L, Wang H, Wang X (2021). Transboundary spread of peste des petits ruminants virus in western China: a prediction model. PLoS ONE.

[11] Xia J, Zheng XG, Adili GZ, Wei YR, Ma WG, Xue XM, Mi XY, Yi Z, Chen SJ, Du W, Muhan M (2016). Sequence analysis of peste des petits ruminants virus from ibexes in Xinjiang, China. Genetic Mol Res.

[12] Munir M, Zohari S, Saeed A, Khan Q, Abubakar M, LeBlanc N, Berg M (2012). Detection and phylogenetic analysis of peste des petits ruminants virus isolated from outbreaks in Punjab, Pakistan. Transbound Emerg Dis.

[13] Abubakar, M., Mahapatra, M., Muniraju, M., Arshed, M. J., Khan, E. U. H., Banyard,A. C., … Parida, S. (2017b). Serological detection of antibodies to peste des petits ruminants virus in large ruminants. *Transboundary and Emerging Diseases*, *64*(2), 513–519. 10.1111/tbed.12392 PMC5347956 26200233

[14] Anderson J, McKay JA, Butcher RN. 1991. The use of monoclonal antibodies in competitive ELISA for the detection of antibodies against Rinderpest and Peste des Petits Ruminants virus. The Proceedings of the Final Research Coordination, in the seromonitering of Rinderpest throughout Africa Phase I, International Atomic Energy Agency, Vienna-Austria, 43–53.

[15] Catley A, Alders RG, Wood JLN (2012). Participatory epidemiology: approaches, methods, experiences. Vet J.

[16] Thrusfield M (2007). Veterinary epidemiology.

[17] Rogan WJ, Gladen B (1978). Estimation of prevalence from the results of a screening test. Am J Epidemiol.

[18] Niyokwishimira A, de D Baziki J, Dundon WG, Nwankpa N, Njoroge C, Boussini H, Bodjo SC (2019). Detection and molecular characterization of Peste Des Petits ruminants virus from outbreaks in Burundi, December 2017–January 2018. Transbound Emerg Dis.

[19] Lawrence KE, Forsyth SF, Vaatstra BL, McFadden AMJ, Pulford DJ, Govindaraju K, Pomroy WE (2017). Cluster analysis of the clinical histories of cattle affected with bovine anaemia associated with Theileria Orientalis Ikeda type infection. N Z Vet J.

[20] Jost C, Mariner JC, Roeder PL, Sawitri E, Macgregor-Skinner GJ. (2007). Participatory epidemiology in disease surveillance and research. Sci Tech Rev. 18293603

[21] IPAPEL, Congo RD. Rapport annuel de l’inspection provinciale de l’agriculture pêche et élevage. Bukavu; 2012. p. 86.

[22] Abubakar, M., Sattorov, N., Manzoor, S., Khan, E. U. H., Hussain, M., Zahur, A. B.,… Wensman, J. J. (2019). Detection of antibodies to peste-des-petits-ruminants virus in the semi-domesticated yak. *European Journal of Wildlife Research*, *65*(6), 88.

[23] Khan HA, Siddique M, Abubakar M, Ashraf M (2008). The detection of antibody against peste des petits ruminants virus in sheep, goats, cattle and buffaloes. Trop Anim Health Prod.

[24] Zahur AB, Irshad H, Hussain M, Ullah A, Jahangir M, Khan MQ, Farooq MS (2008). The epidemiology of peste des petits ruminants in Pakistan. Rev Sci Tech.

[25] Zahur, A. B., Shahzad, C., Shahzad, C., Shahzad, C., Shahzad, C., Shahzad, C., … Kashmir,A. (2014). Epidemiological analysis of Peste des Petits Ruminants (PPR) outbreaks in Pakistan. *Journal of Biosciences and Medicines*, *2*(06), 18.

[26] Saritha G, Shobhamani B, Sreedevi B (2014). Seroprevalence of peste des petits ruminants in pastoral small ruminants with special reference on sensitivity to age and agro-climatic zones (India). Anim Sci Report.

[27] Nikmal Azizi AF. (2010). Peste des petits ruminants in Afghanistan. Ministry of Agriculture, Irrigation, and Livestock, Kabul, Afghanistan. https://oiebulletin.com/?panorama ppr control and eradication programme in afghanistan 2. 2010.

[28] Noman MA, Shaikat A, Nath B, Shil S, Hossain M (2011). Incidence and modulating effects of environmental factors on infectious diseases of Black Bengal goat in Cox’s Bazar district of Bangladesh. YYU Veterier Fakultesi Dergisi.

[29] Ishaque SM (1993). Sheep management systems. Sheep Production in Pakistan.

[30] Selvaraju G (2014). Epidemiological measures of causal association between Peste Des Petits Ruminants (PPR) and its determinants in small ruminants. Int J Dev Res.

[31] Al-Majali AM, Hussain NO, Amarin NM, Majok AA (2008). Seroprevalence of and risk factors for peste des petits ruminants in sheep and goats in Northern Jordan. Prev Vet Med.

[32] Radostits OM, Gay CC, Hinclcliff KW, Constable PO. (2007). Veterinary Medicine: A text book of the disease of cattle, sheep, pigs, goat and horses. 10 ed. London, Saunders, pp: 1094–1110.

[33] Gebre T, Deneke Y, Begna F (2018). Seroprevalence and Associated Risk Factors of Peste Des Petits Ruminants (PPR) in Sheep and goats in four districts of Bench Maji and Kafa Zones, South West Ethiopia. Global Vet.

[34] Kgotlele T, Torsson E, Kasanga CJ, Wensman JJ, Misinzo G (2016). Seroprevalence of Peste Des Petits ruminants Virus from SamplesCollected in different regions of Tanzania in 2013 and 2015. J Veterinary Sci Technol.

[35] Prajapati M, Shrestha SP, Kathayat D, Dou Y, Li Y, Zhang Z (2021). Serological investigations of Peste Des Petits ruminants in cattle of Nepal. Veterinary Med Sci.

